# DDM1-Mediated TE Silencing in Plants

**DOI:** 10.3390/plants12030437

**Published:** 2023-01-18

**Authors:** Ruth Y. Akinmusola, Catherine-Axa Wilkins, James Doughty

**Affiliations:** Department of Life Sciences, University of Bath, Bath BA2 7AY, UK

**Keywords:** DDM1, transposons, small RNAs, RdDM, DNA methylation, histone modifications, chromatin, heterochromatin, TE silencing

## Abstract

Epigenetic modifications are indispensable for regulating gene bodies and TE silencing. DECREASE IN DNA METHYLATION 1 (DDM1) is a chromatin remodeller involved in histone modifications and DNA methylation. Apart from maintaining the epigenome, DDM1 also maintains key plant traits such as flowering time and heterosis. The role of DDM1 in epigenetic regulation is best characterised in plants, especially arabidopsis, rice, maize and tomato. The epigenetic changes induced by DDM1 establish the stable inheritance of many plant traits for at least eight generations, yet DDM1 does not methylate protein-coding genes. The DDM1 TE silencing mechanism is distinct and has evolved independently of other silencing pathways. Unlike the RNA-directed DNA Methylation (RdDM) pathway, DDM1 does not depend on siRNAs to enforce the heterochromatic state of TEs. Here, we review DDM1 TE silencing activity in the RdDM and non-RdDM contexts. The DDM1 TE silencing machinery is strongly associated with the histone linker H1 and histone H2A.W. While the linker histone H1 excludes the RdDM factors from methylating the heterochromatin, the histone H2A.W variant prevents TE mobility. The DDM1-H2A.W strategy alone silences nearly all the mobile TEs in the arabidopsis genome. Thus, the DDM1-directed TE silencing essentially preserves heterochromatic features and abolishes mobile threats to genome stability.

## 1. Introduction

Transposable elements (TEs) are genetic elements that are mobile within a genome or have lost their mobility directly or through an RNA intermediate [1]. TEs influence genome diversity through their mobility, replication and high copy numbers in almost all eukaryotes [2]. There are class I TEs and class II TEs based on their structure and transposition mechanisms. Class I TEs or retrotransposons transpose through an RNA intermediate in a copy-and-paste mechanism. Thus, the element undergoes reverse transcription before integrating into the nuclear genome, and old copies persist [3]. In contrast, class II TEs are DNA transposable elements that transpose in a cut-and-paste mechanism [2]. This cut-and-paste mechanism suggests that the number of elements does not increase in the genome. However, DNA repair mechanisms can repair the excision site through homologous recombination to regenerate the original element [2,4]. Both classes have autonomous and non-autonomous elements. Autonomous elements possess open reading frames that encode the proteins required for transposition. In contrast, non-autonomous elements do not encode any proteins, and they require the proteins provided by autonomous elements for their transposition [2]. The changes caused by TEs have played a significant role in plant evolution [5]. TE acquisition correlates with genome size. Plant species such as maize and barley with big genome sizes have higher proportions of TEs (above 85% of TEs), whereas *Arabidopsis thaliana* and *Brachypodium distachyon* with smaller genomes contain low numbers of TEs (20% to 30%) [6,7]. 

TEs can be transmitted through horizontal transfer between genomes [8,9]. Following their incorporation into the genome, TEs can cause different genetic variations. TE insertions drive genome evolution through reprogramming expression patterns, gene transpositions and regulatory networks [5,10]. Some TEs are vital components of heterochromatin to maintain chromosome stability and heterochromatic features [11]. However, the deleterious activities of TEs during their insertion and mobility in the genome make them parasitic DNA fragments and a target for TE silencing. The degree and speed at which these transpire can significantly vary depending on the TE activity and copy number [5]. Diverse epigenetically derived pathways regulate TE expression, mobility, replication and recombination in plants [12,13]. These pathways repress TE activity with different strategies, such as DNA methylation, histone modifications and RNA interference (RNAi) [11,12,14,15,16]. DNA methylation is more ubiquitous than the other epigenetic modifications in the plant genome [12,17]. It epigenetically defines and maintains several processes, such as genomic imprinting, stress responses and seed development [12,17,18,19]. 

The bulk of plant DNA methylation also occurs in the regions of the genome populated by TEs. This is in different sequence contexts (CG, CHG and CHH (where H = A, T or C)), and each sequence context is catalysed by a distinct methyltransferase in plants [12]. Genic methylation in the CG context is maintained by DNA METHYLTRANSFERASE 1 (MET1), a homolog of the mammalian DNA METHYLTRANSFERASE 1 (DNMT1) [20]. CHG methylation is maintained by CHROMOMETHYLASE 3 (CMT3), a plant-specific CMT family of methyltransferases, with a conserved CHROMO domain [21]. CHH methylation is maintained by another member of the CMT family, CHROMOMETHYLASE2 (CMT2) [11]. Mutations in both the RNA-directed DNA pathway (RdDM) and the DECREASE IN DNA METHYLATION 1 (DDM1) pathways lead to progressive changes in MET1, CMT2 and CMT3 DNA methylation marks [12]. However, the RdDM and DDM1 roles in TE silencing are regulated by different mechanisms, and their distinct roles may lead to a complex regulatory interplay at the whole-genome scale [13,22]. The RdDM utilises small RNAs to mediate DNA methylation in plants for TE and gene body silencing [12]. The chromatin remodeller, DDM1, rarely silences genes but is mainly involved in TE silencing in inaccessible heterochromatic regions [11,23]. 

This review focuses on the DDM1 TE silencing machinery in plants. We first provide an overview of the biological significance of this gene and then examine how DDM1 acts both synergistically and distinctly from the RdDM. 

## 2. DDM1 in Trait Regulation

### 2.1. DDM1 Participates in a Broad Range of Plant Traits

**Figure 1:**
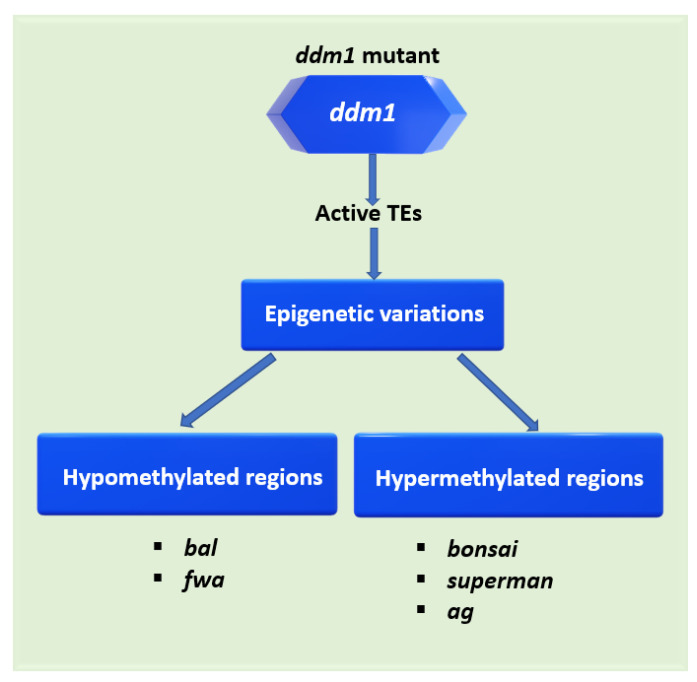
*ddm1*-induced defects can introduce epigenetic variations typically not observed in the wild-type. Without an intact *DDM1* gene, active TEs in the genome can transpose into or change methylation status in the promoters or regions near essential coding genes to disrupt gene expression [24,29,30,33,34,35]. These unlinked loci can be hypermethylated or hypomethylated, leading to abnormal phenotypes.

DDM1 is involved in arabidopsis traits such as hybrid vigour, heterosis, flowering time, plant height, DNA damage responses, biotic stresses and sensitivity to radiation [24,25,26]. Arabidopsis *ddm1* mutants display many pleiotropic traits. These include delayed flowering, dwarfism, reduced hybrid vigour, sensitivity to salt stress, gamma and UV radiation sensitivity, the restoration of hygromycin resistance and global demethylation in all sequence contexts [26,27,28]. The *ddm1*-induced abnormalities are attributed to some unlinked genomic loci, including TEs, other repeat elements and even protein-coding genes (Table 1). These may be directly related to other plant traits, such as elevated pathogen resistance in *ddm1*-induced *bal* mutants [29]. The *fwa*, *superman* and *agamous* (*ag*) phenotypes in *ddm1* mutants are also displayed in *met1* mutants, suggesting that these three phenotypes are also under the control of genic CG methylation [20,30]. However, the *bal* and *bonsai* syndrome exclusively depend on *ddm1* [29,30]. Therefore, DDM1 is required for the genetic stability of many arabidopsis traits. Its mutation may cause the misexpression of other genes to create different epialleles (Figure 1, Table 1). The abnormal flowering and dwarfing phenotypes underlying the expression of these epialleles are normally required for reproductive fitness, and these could affect hybridisation events when stably inherited in both immediate and future generations with defective DDM1 alleles [31,32]. Thus, DDM1 acts as a transgenerational epigenetic guardian of the genome modulating the formation of undesirable epialleles potentially affecting genetic diversity. 

A panel of *ddm1* mutants in arabidopsis, rice and maize have been used to elucidate their roles in plant development. There are two *DDM1* homologs in rice (*OsDDM1a* and *OsDDM1b*), maize (*ZmDDM1a* and *ZmDDM1b*) and tomato (*SlDDM1a* and *SlDDM1b*), but the double mutants of these genes in their respective genomes typically result in embryo lethality [36,37,38]. The sterile plants produce embryos with slower cell proliferation and abnormal polyploid cells, suggesting extreme delays in cytokinesis and mitosis, thus, limiting the studies on these mutants [39]. In contrast, the single mutants in these plants produce viable embryos with pleiotropic developmental phenotypes starting from their initial generation [36]. However, it is possible to propagate homozygous *ddm1* alleles for more generations in *A. thaliana*, as the plant only has a single copy of the *DDM1* gene, which is not lethal when knocked out [37]. 

The high tolerance of arabidopsis to *DDM1* gene disruption has been linked to its lower genomic TE content, unlike rice, maize and tomato genomes with significantly higher TE content [36,37,40]. The presence of two *DDM1* orthologues in rice and the other complex genomes make their immediate generations sterile and more sensitive to genome-wide disruptions in methylation patterns [37]. These strongly suggest that the *DDMI* gene role in the plant methylome is not just required for TE silencing but also for embryogenesis and the maintenance of trait diversity. Further characterisation of these two *DDM1* orthologues using the methylome, transcriptome and small RNAs and cell imaging may unravel the conserved and distinct functions of the two genes. This will shed more light on the evolution and epigenetic variation in *DDM1* functions in both natural populations and different plant lineages. 

### 2.2. DDM1 Is Genetically Distinct from DDM2 

DDM1 and DDM2 (DECREASED DNA METHYLATION 2) are required for the maintenance of genome integrity, and their mutations result in global demethylation [20,24]. *DDM1* and *MET1* gene mutations are often used to study heterochromatin-related methylation patterns. However, their roles in DNA methylation are quite distinct [11,24,41]. The *DDM2* locus in plants encodes MET1, which is preferentially involved in CG methylation. In contrast, DDM1 is a chromatin remodeller involved in DNA methylation and histone methylation in CG and non-CG contexts [11,20,42]. MET1 can also act downstream of siRNA-dependent but DDM1-independent TE silencing pathways for genic CG methylation in the RdDM pathway [12,41]. However, the DDM1-MET1 pathway can be siRNA-independent, suggesting some overlapping roles between the two pathways. The demarcation between their similarities and contrasts lies in histone modifications and the interactions with the other TE and gene silencing pathways, such as the RdDM [11].

Arabidopsis strains with defective DDM1 and DDM2 alleles are fertile but with some pronounced morphological abnormalities following inbreeding. Specifically, a reduced canopy size, altered leaf shapes and delayed flowering dates [20]. However, these defects are more evident in *ddm1* mutants, tending towards sterility in the ninth inbred generations [43]. Furthermore, compared to *met1* mutants, unique phenotypes are shared across *ddm1* mutants [20,26,28,44]. These include a decreased heterosis level, DNA damage response and increased branching [25,26,43]. The chromatin structure and nucleosome accessibility define the DDM1 and MET1 plant traits. Some DDM1-specific phenotypes, such as DNA damage response, have been linked to altered chromatin structure. In contrast, the other phenotypes shared by the two genes are associated with global changes in cytosine methylation patterns [26]. 

**Figure 2:**
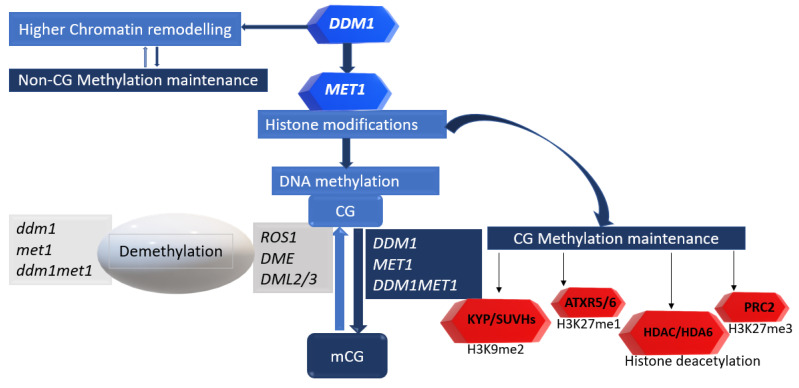
*DDM2* (*MET1*) can act downstream of genic *DDM1* activity in the CG context. This could be by direct histone modifications, methylation with specialised histone methyltransferases or deacetylation of histone tails with HDAC proteins, such as HDA6. The dark-blue arrow represents the gene products or proteins that can establish the CG methylation marks (the deep blue and red boxes on the right), while the light-blue arrow represents the genes that can remove these methylation marks (grey boxes on the left). The CG methylation marks can be removed by demethylases (*ROS1*, *DME* or *DMLs*) or by repressing *DDM1* and *MET1* (grey boxes on the left). The POLYCOMB REPRESSIVE COMPLEX 2 (PRC2) has a histone methyltransferase activity involved in histone H3 lysine 27 trimethylation (H3K27me3). ATXR5 and ATXR6 are plant-specific H3K27me1 methyltransferases involved in female germline development. *ROS1*: *REPRESSOR OF SILENCING 1*, *DME*: *DEMETER*, *DML*: *DEMETER-LIKE*, SUVHs: SET DOMAIN-CONTAINING PROTEIN 3, KYP: KRYPTONITE, ATXR5/6: ARABIDOPSIS TRITHORAX-RELATED PROTEIN 5/6, HDAC: HISTONE DEACETYLASE, mCG: CG methylation marks, and CG: removed CG methylation marks. Adapted from [24,45].

On the genome scale, *ddm2* (*met1*) defects acting downstream of *ddm1* have been attributed to the global demethylation patterns across several genomic loci (Figure 2). Many TEs gain H3K27me3 when hypomethylated by *ddm1,* suggesting a genome-wide antagonism between H3K27me3 and DNA remethylation [45]. Similarly, H3K27me1 also co-localises with DNA methylation with *ddm1* loss [46]. In contrast, decreased H3K9 and DNA demethylation at chromocenters are observed in *ddm1* and *met1* mutants [24]. H3K9 methylation is a major histone modification regulating DNA methylation in *Arabidopsis* [46]. In addition, histone deacetylases, such as HDA6, also interact with MET1 in CG methylation, requiring an intact DDM1 [24,47]. Furthermore, there is a clear link between global DNA methylation and meiotic recombination rates in the euchromatin and heterochromatin. The *ddm1*/*ddm2* mutant roles in these are distinct in the genome, with DDM1 having higher impacts in the heterochromatin, a gene-poor region with lower recombination rates [24,45,48]. 

## 3. DDM1 in the RdDM Context

### 3.1. DDM1 Collaborates with RdDM in Whole-Genome TE Silencing

The RNA-directed DNA Methylation Pathway (RdDM) and DDM1 are involved in DNA methylation at all sequence contexts (CG, CHG and CHH) [12]. The differences between the two pathways are summarised in Table 2. The RdDM pathway is an evolutionarily conserved DNA methylation mechanism unique to plants. Its operational machinery relies on two plant-specific homologs of RNA polymerase II (Pol IV and Pol V) [49,50]. The Pol IV branch of the pathway is involved in the recruitment of short non-coding RNAs, and these are converted into double-stranded RNA by RNA-dependent RNA polymerase 2 (RDR2) [51,52]. The double-stranded RNA molecules are later processed by DCL3 (DICER-LIKE 3) into 24 nucleotide RNAs [53]. The RNA Pol V branch acts with the other components to load these small RNAs into an Argonaute complex, usually AGO4 or AGO6 [54]. The RdDM pathway is involved in DNA methylation at unmethylated loci in the genome (*de novo* RdDM) and established methylated loci (canonical RdDM) [49]. The differences between canonical and non-canonical RdDM are the source and type of the siRNA scaffold for DNA methylation. This is usually the 24nt sRNAs specifically required for canonical RdDM and the 21–22nt sRNAs that function in non-canonical RdDM and other gene silencing pathways such as RNAi (RNA interference) [12]. Thus, the non-canonical RdDM pathway has evolved as a *de novo* mechanism to protect against the accidental activation of novel TE insertions [12].

Recent evidence has suggested that the DDM1 and RdDM pathways have evolved independently in plants as a double insurance mechanism for DNA methylation [49,55]. So far, only mutants in the RdDM pathway are known to block *de novo* DNA methylation in plants [51,56]. The RNA-dependent RNA polymerase 6 (RDR6) branch of the RdDM pathway induces the *de novo* DNA methylation of novel TE insertions or newly activated TE sequences [12]. *DDM1* mutation can also activate RDR6-RdDM *de novo* DNA methylation, suggesting the reactivation of some TEs specifically methylated by DDM1 [55,57]. The first *de novo* DNA methylation pattern recorded in *ddm1* mutants relies on the H3K9 methyltransferase, KRYPTONITE (KYP) and CMT3, a methyltransferase [16,57,58]. CMT3 and CMT2 act downstream of the RdDM pathway in the maintenance of CHG and CHH methylation, respectively [12]. Both methyltransferases act downstream of DDM1 to methylate DNA in the H3K9me2-marked heterochromatin [59,60]. However, unlike *ddm1* and RdDM mutants, *cmt2*, *cmt3* and *cmt2cmt3* mutants alone do not result in the demethylation of several TEs due to the dominance of DDM1 and RdDM methylation in the genome [11,61]. DDM1 loss results in a methylation switch from CMT2 to the RdDM to enforce CHH methylation in the borders of long TEs [55]. The CMT2 to RdDM switch has been proposed to be a buffer mechanism against the loss of some essential chromatin marks in the *ddm1* mutant [55]. Since the DDM1 pathway is not a by-product of the RNAi Post-transcriptional Gene Silencing (PTGS) pathway and does not utilise small RNAs, the DDM1 and the RdDM pathways have evolved independently to establish different chromatin marks in the genome. 

**Figure 3:**
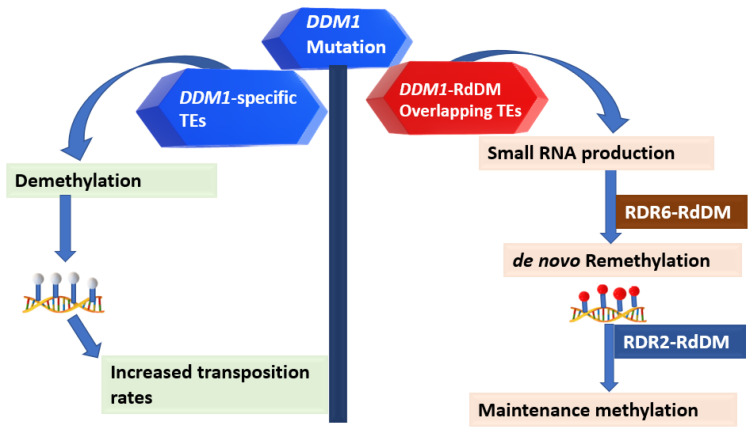
A model differentiating TEs silenced by the DDM1 and RdDM pathways. *DDM1* gene mutation leads to global demethylation in all sequence contexts. Only the TEs targeted by both pathways are remethylated by the RdDM pathway. Red dots indicate methylated sequences, and white dots indicate demethylated sequences in the CG, CHG and CHH contexts.

The DDM1 and RdDM TE silencing pathways are mutually inclusive in wild-type conditions at the whole-genome scale [12,22]. However, *ddm1* mutants have more severe phenotypic abnormalities than RdDM mutants [30,37,39]. Apart from the distorted methylation patterns in a small number of unlinked loci, such as some protein-coding genes, *DDM1* mutations predominantly cause uninhibited torrents of TE transposition that worsen following several generations of inbreeding [62]. This strongly suggests that DDM1 exclusively targets mobile TEs, and these are distinct from those targeted by the RdDM. Two distinct models distinguish the TEs silenced by DDM1 and the RdDM (Figure 3). First, a subset of the TEs activated following *ddm1* inactivation are sensed as novel TE insertions, triggering *de novo* re-methylation by the RDR6-RdDM pathway. Both pathways can jointly silence these TEs, but only a minority of the re-silenced TEs are mobile [22,55]. Secondly, the other mobile TEs activated in *ddm1* mutants cannot be re-methylated by the *de novo* RdDM pathway [22,37,63]. This is because the RdDM pathway does not independently silence mobile TEs with protein-coding regions and cannot stop their transposition [11].

The few TEs methylated by both pathways can be in the same family. For instance, Gypsy elements possessing a high proportion of sRNA in their whole sequence require both the DDM1 and RdDM pathways for their methylation [11]. However, the choice of silencing mechanism depends on the heterochromatic features of the internal TE sequences. DDM1-mediated methylation targets internal TE features, whereas the RdDM machinery can only methylate the long TE edges [11,55,64]. The mobile TEs silenced by DDM1 are hallmarked by protein-coding regions essential for increased TE activity and transposition [22]. *DDM1* mutations in arabidopsis accrue heritable epialleles leading to increased TE transposition rates [24,30]. Even in the ninth generation of inbred *ddm1* mutants, the RdDM pathway cannot silence these DDM1-specific transposition TEs [30,65]. Thus, the role of DDM1 in TE silencing is genetically independent and does not rely on the RdDM feedback loop. However, how the RdDM methylation marks are diluted out in the ninth of generation *ddm1* mutants remains to be elucidated.

### 3.2. The Collaboration between DDM1 and the RdDM Is Linked to TE Activity and Small RNA Dynamics in the Genome

More than any other epigenetic pathway combination, such as RNA interference plus RdDM, DDM1 and RdDM pathways synergistically mediate the epigenetic silencing of nearly all TEs in the plant genome [11,13,22]. A typical TE lifecycle begins from genome invasion to proliferation, degeneration and, eventually, TE senescence [77,78]. This is further complicated by the TE silencing status, ranging from *de novo* methylation to maintenance and eventual release from epigenetic silencing [13]. In addition to the main TEs, TE relics are shorter but need to be silenced as they are preferentially close to protein-coding genes [79]. TE relics are the TEs that have either lost their autonomous transposition ability or the capacity to promote the transposition of other elements [13,22]. The RdDM pathway targets newly inserted TEs, short active TEs, the edges of long active TEs and TE relics enriched in the chromosome arms [11,55,64]. The DDM1 pathway preferentially silences long heterochromatic TEs [11,42]. Based on the TE landscape, RdDM-mediated activity is predominant in the euchromatin, whereas DDM1 acts in the heterochromatin [80]. RdDM-DDM1 dynamics at these genomic locations are adapted to initiate and enforce TE silencing at all stages of the TE life cycle. 

In *A. thaliana*, and other plant genomes, TEs are more densely arranged in heterochromatic regions, whereas TEs found in the euchromatin are typically evenly distributed within or close to genes [6]. Though the RdDM pathway partly operates in the heterochromatin context, most heterochromatic TEs require DDM1 for silencing [11]. However, most *ddm1* mutants have increased recombination frequency in the euchromatin than at the centromeres [48,81,82]. This is consistent with the normal observation of recombination frequency in both the hotspots and cold spots of the genome, strongly suggesting that DDM1 also acts in euchromatic regions [83]. TE silencing can be triggered by transposon-derived sRNAs, which could be siRNAs or miRNAs [84]. SiRNA-producing euchromatic TEs with low GC content in gene-rich regions are methylated by the RdDM pathway, whereas the siRNA-producing heterochromatic TEs are methylated independently of siRNA [14]. In tomato *ddm1* mutants, there is a loss of mGGs and mCHGs in heterochromatic TEs; however, some euchromatic TEs also have depleted mCHH [37]. Thus, siRNA production and TE type also shape the methylation patterns in euchromatin and heterochromatin.

In plants, DDM1 and the RdDM mediate TE methylation to prevent their transposition [11]. Their synergistic action also reinforces the epigenetic and genic expression of many plant phenotypes, such as flowering time, biotic and abiotic stresses [12]. The sRNA-producing loci are the main methylation sites for the RdDM pathway and other Post-transcriptional Gene Silencing (PTGs). For instance, siRNA accumulation in TEs such as *Athila* prevents transposition and promotes transgenerational TE silencing [85]. Furthermore, a reduction in 24nt-siRNA abundance may be partly linked to hypomethylation at some RdDM-associated genomic loci [86]. Small RNA production is also influenced by the TE family, TE location, structure and copy number [11,87]. In contrast to the RdDM pathway and other PTGS mechanisms, DDM1-directed methylation is not correlated to sRNA abundance as the DDM1 machinery silences siRNA-independent regions [11,88]. Nevertheless, siRNA production does not always culminate in TE silencing as the CACTA TE family in rice produces microRNAs to suppress TE silencing [84]. These microRNAs in the *miR820* gene family preferentially target *OsDRM2*, one of the key RdDM components [84]. Thus, the DDM1 pathway has not only evolved as a mechanism for silencing some sRNA-deficient TEs but also some sRNA-producing TEs that can escape DNA methylation by the RdDM pathway. Though the RdDM pathway can autonomously initiate and maintain TE silencing, it is ineffective in inaccessible heterochromatin and cannot silence protein-coding TEs with transposition ability [22]. 

## 4. DDM1 Is More Active in the Heterochromatin

### 4.1. DDM1 Is Required to Preserve Heterochromatic Features, and This Is Independent of the RdDM Pathway

DNA is typically packaged in nucleosomes—the structural and functional unit of chromatin. Structurally, each nucleosome contains two of each of the core histones: H2A, H2B, H3 and H4, combined to form a protein octamer wrapped by approximately 146 bp of DNA [76]. Each chromosome in the genome consists of millions of nucleosomes joined by a linker stretch of around 20 bp situated between them [89]. Histone H1 serves as a linker to bind nucleosomes and their intervening linker DNA [90]. The linker histone H1 and the H2A variants are the most divergent histone family, directly affecting the nucleosome biochemical properties [91,92]. These effects include chromocenter condensation, DNA methylation patterns and histone post-transcriptional modifications [11,92]. Heterochromatin refers to the condensed chromatin regions enriched with transposable elements and other repetitive elements such as 5S ribosomal RNA gene repeats [41]. In contrast, euchromatin is more accessible with higher transcriptional activity due to the prevalence of protein-coding genes. Thus, the fundamental chromatin structure is not just crucial for driving genome organisation but provides insights into TE dynamics and chromatin accessibility. 

Chromatin structure is highly compact but dynamic, and transcriptional activity varies between the gene-rich euchromatin and the TE-rich heterochromatin [93]. DNA methylation occurs in both TEs and gene bodies in all sequence contexts. However, TE methylation could be intragenic, with deleterious effects on gene expression. To maintain proper TE silencing, the default tight structure of the heterochromatin must be accessible to RNA polymerases, transcription factors and other nuclear factors [11]. This is accomplished through two main strategies: (i) via chromatin remodellers such as the DDM1 protein modifying DNA–histone interactions utilising ATP hydrolysis to expose the underlying DNA to enzymes such as RNA polymerases [94] and (ii) via the enzymatic modification of DNA and histone residues [95]. The mechanisms underlying these two processes are interconnected and reversible as the decompressed heterochromatin returns to its compact state [93]. There are four classes of chromatin remodelling complexes in plants, and among these, DDM1 cooperates with the others, such as DEFECTIVE IN RNA-DIRECTED DNA METHYLATION 1 (DRD1), to maintain higher-order chromatin structures [11]. However, the exact mechanisms of ‘activating the remodelling’ or ‘deactivating the remodelling’ functions of the other chromatin remodelling complexes are unclear.

The production of siRNAs, typically originating from regions of euchromatin in wild-type plants, becomes activated in regions of heterochromatin in *ddm1* mutants [37]. An intact DDM1 protein makes the heterochromatin inaccessible for methyltransferases and other enzymes responsible for siRNA synthesis [68]. The RdDM may spread to the heterochromatin in many ways. First, by the expansion of siRNA production to the heterochromatin. This allows RNA polymerases, such as MET1, CMT2 and CMT3, to methylate heterochromatic sequences [23]. The diversity of these enzymes is rooted in distinct sequence contexts. CMT2 and CMT3 mediate non-CG methylation, and MET1 mediates CG methylation in the heterochromatin. The RdDM facilitates the action of these genes in methylating the pericentromeric regions via *DRM2* [11]. The Pol IV activity of the RdDM machinery can also be directed to other regions with the action of other chromatin-modifying pathways, such as CLASSY (CLSY) proteins, SAWADEE HOMEODOMAIN HOMOLOG 1 (SHH1) and DEFECTIVE IN RNA-DIRECTED DNA METHYLATION 1 (DRD1) [12,55,96,97]. DRD1 interacts and acts downstream of DDM1, and it is an integral part of the DDR complex consisting of DRD1, DEFECTIVE IN MERISTEM SILENCING 3 (DMS3) and RNA-DIRECTED DNA METHYLATION 1 (RDM1) [98]. This complex also facilitates Pol IV recruitment to heterochromatic sites; however, its interactive network with DDM1 still needs to be better understood [99]. DDM1 loss promotes a compromised heterochromatin [68]. Therefore, the broad chromatin features mark the heterochromatin boundary between DDM1 and the RdDM. Thus, in wild-type conditions, DDM1 is antagonistic to the RdDM pathway in compact heterochromatin [72]. However, it is unclear whether this antagonism is broadly conserved in all plant genomes.

### 4.2. DDM1 Activity in the Heterochromatin Extends to the Histone Core to Silence the Majority of Mobile TEs

DNA methylation is smoothly established and maintained in nucleosome-free DNA [23]. Histone H1, a nucleosome linker, is the first immediate barrier to heterochromatin. Its inactivation in arabidopsis partially rescues the *ddm1* phenotype [11]. In *ddm1* and *h1* mutants, siRNA biogenesis, RdDM pathway and methyltransferases are redirected from their euchromatic targets to the heterochromatin [11,14,80]. However, this redirection is only to a smaller proportion of the heterochromatin, preferentially the H1 and H3K9me2 regions. This indicates that the RdDM pathway alone cannot methylate entire regions of compromised heterochromatin [11,80]. The TEs requiring H1 activity in the heterochromatin are different from RdDM targets in the euchromatin [11]. They are short, autonomous AT-rich TEs and TE remnants devoid of protein-coding regions [80]. Thus, H1 restricts RdDM access to the long, GC-rich TEs with coding regions, which the CHROMOMETHYLASE (CMT) pathway also methylates in *ddm1* mutants [55,80,100]. The dynamics in H1 activity also have profound effects on gene bodies, as dispersed heterochromatin promotes genic methylation [100]. Furthermore, the loss of H1 in heterochromatic regions can impede chromocenter condensation, leading to heterochromatin H1, H2 and H3 domains physically interacting with the DDM1 protein [76,80].

**Figure 4:**
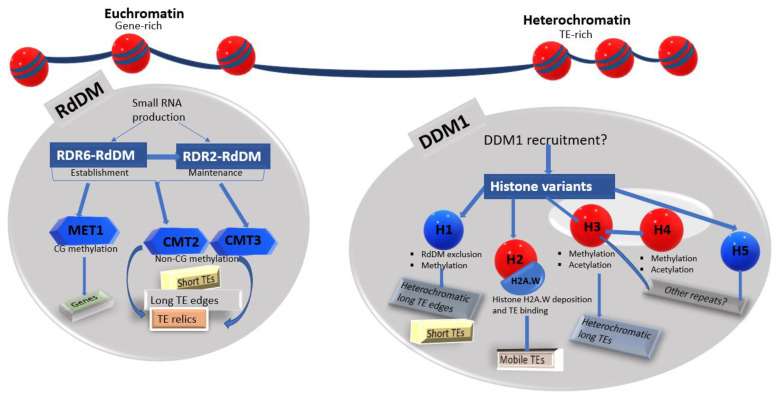
A model for the role of DDM1 in maintaining TE silencing in *Arabidopsis thaliana*. The chromatin environment shapes the epigenetic landscape for TE silencing. RdDM is predominant in the euchromatin and excluded from the heterochromatin by DDM1 via histone H1 [11,23]. DDM1 is required to maintain the heterochromatin and silence TEs via different histone variants. DDM1-mediated H2A.W deposition exclusively silences mobile TEs [22]. Some immobile TEs are also silenced by DDM1 in the heterochromatin [11]. RdDM: RNA-directed DNA methylation, RDR2: RNA-DEPENDENT RNA POLYMERASE2, RDR6: RNA-DEPENDENT RNA POLYMERASE6, MET1: DNA METHYLTRANSFERASE1, CMT2: CHROMOMETHYLASE2, CMT3: CHROMOMETHYLASE3 and TE: Transposable element.

In arabidopsis, the persistence and enrichment of methylated TEs and gene sequences in *h1* strongly suggest other silencing mechanisms [100]. The DDM1-mediated access for heterochromatin methylation is not restricted to only H1, as increasing evidence shows that DDM1 can also remodel the H2 domain to methylate sequences [76,80]. The combined loss of H1 and H2A.W increases heterochromatin accessibility to a greater degree than H1 loss alone [22]. In addition, H1 and H2 interact to prevent excessive H1 incorporation, suggesting rivalry for linker DNA binding [76]. DDM1 directly binds to H2A.W, a variant of histone H2A with two distinct regions, and mobile TEs are silenced by H2A.W deposition into the heterochromatin [22]. Both mechanisms act independently of H3K9me2 and are sufficient to silence most heterochromatic TEs, including the loss of H3K9me2 activity [22]. The DDM1-mediated H2A.W deposition silences approximately 88% of the identified mobile TEs in the arabidopsis genome. Additionally, the loss of H2A.W is predominant in the TE bodies compared to the TE fragments, as DDM1 preferentially mediates H2A.W deposition in the TE protein-coding regions [22]. Since TE fragments cannot regain mobility, the DDM1-H2A.W strategy has distinctively evolved from the H1 strategy to silence mobile TEs and control their transposition (Figure 4).

H3K9me1/2, H3K27me1 and H3K4me1/2/3 are the other epigenetic modifications associated with DDM1 activity in heterochromatic modifications. The methylation marks in these heterochromatic regions could be positive or repressive for transcription. For example, H3K27me3 is associated with the polycomb repressive complex (PRC) pathway to repress many developmental genes transcriptionally [45]. The H3K4me1/2/3 displays a positive epigenetic signature in enhancers and promoters [101]. The RNA polymerase IV component of the RdDM pathway is often recruited via SHH1 to target H3K9 methylation. This suggests that DDM1 and the RdDM pathway are interconnected with varying effects on the other heterochromatic features. In addition to a reduction in global DNA methylation, H3K9 methylation is depleted in *ddm1* mutants [72]. Furthermore, the loss of H3K9 methylation is permanent in *ddm1* mutants even with restored DDM1 expression [22]. *DDM1* mutation in rice is also associated with repression at H3K4me3, H3K9me2, H3K27me3 and H3k9ac methylation marks [72,102]. In addition to Histone H3 repressive marks, DDM1 promotes H4K16 deacetylation [24]. However, the DDM1 TE silencing mechanism in the histone H3 and H4 domains is H2A.W-dependent [22]. This dependency is likely to operate in a feedback loop involving the CHROMOMETHYLASES (CMTs) in non-CG methylation. Nevertheless, the hierarchy of the processes involved in DDM1-led deacetylation is still unclear, but DNA methylation is presumed to occur first [24]. 

Thus, the role of DDM1 in maintaining the heterochromatic state is associated with various chromatin features, including DNA methylation, DNA acetylation, histone activity, TE mobility and other silencing pathways (Figure 4). DDM1 and the RdDM are the major TE silencing pathways in plants, and they separately silence distinct TEs in different chromatin environments [11]. The compact heterochromatin, a TE-rich region, requires DDM1 to silence heterochromatic and mobile TEs by remodelling different histone variants [11,22,23,100,103]. RdDM depends on small RNA production and is predominant in the euchromatin to methylate gene sequences and TEs situated close to genes (Figure 4). Histone H1, under the action of DDM1, prevents RdDM and other methylation pathways from accessing the compact heterochromatin [11,23]. H2A.W deposition also excludes RdDM activity from the heterochromatin but only in the presence of H1 [22,76]. Notwithstanding, the DDM1-mediated H2A.W silencing activity is quite distinctive, as approximately 40% of the genome-wide TEs are hypomethylated in *ddm1* mutants with depleted H2A.W levels [22]. Most of these are potentially mobile or transposition TEs, indicating that the DDM1-H2A.W strategy is the primary mode for silencing mobile TEs [22]. None of the other DNA methylation and histone modification pathways silences heterochromatic TEs and controls TE transposition to the degree achieved by DDM1 [22,104].

## 5. Conclusions and Outlook

The role of DDM1 in TE silencing varies with the TE context, depending on the proximity to genes and TE mobility [11,22]. Disruptions to the mouse DDM1 homologue (Lsh) result in a plant-like DDM1 methylation phenotype suggesting some conservation of mechanism and function [105]. Still, there are gaps in understanding DDM1 pathway differences in both organisms, especially the functional impacts on their developmental phenotypes. Evaluating more *ddm1* mutants, especially in an array of other plant genomes, may provide a better understanding of the complex roles played by DDM1 in epigenetic regulation. Do the differences in plant tissues or cell types play a significant role in the diversity of DDM1 DNA methylation or histone modifications? How exactly are the DDM1-mediated modifications transmitted through all the stages of cell division over several generations? 

Further studies may help unravel the molecular network of the epigenetic switches involved in DDM1-directed TE silencing. Specifically, by quantifying the DDM1-mediated DNA methylation roles to the DDM1-directed histone modification roles and their crosstalk. More insights are also needed to dissect how DDM1 interacts with the other factors, such as H3K9 methylation and the DDR complex in chromatin modification. Such investigations would resolve more nodes in elucidating a well-characterised DDM1 pathway.

## Figures and Tables

**Table 1:** Epialleles induced by *DDM1* null mutations.

Loci	Induced Defect	Other Genes Involved	Reference
*bal*	Dwarfing	NBS-LRR-class disease-resistance gene cluster	[29]
*fwa*	Late flowering	SINE (Short interspersed nuclear element) in the *FWA* promoter region	[24,33]
*bonsai*	Dwarfing Late flowering	*BONSAI* gene flanked by long interspersed nuclear element (LINE)	[30]
*superman* (*sup*)	Abnormal flower development	*DECREASED DNA METHYLATION 2* (*DDM2*)	[34,35]
*agamous* (*ag*)	Abnormal flower development	*DECREASED DNA METHYLATION 2* (*DDM2*)	[34]

This table shows the epialleles underlying the abnormal phenotypes in *ddm1* mutants.

**Table 2:** The key features distinguishing DDM1- and RdDM-mediated epigenetic changes.

	RdDM	DDM1
In both animals and plants	Unique to plants [12,66].	In both, with a DDM1 homologue in mammals [11,23,39,67]
Heterosis and embryo lethality	RdDM mutants are viable and can reproduce even when inbred with no significant effects on plant heterosis [12,19].	Yes, *DDM1* mutation may possess lethal effects on the embryo, which is severe in double mutants. Inbred *ddm1* mutants generally have reduced heterosis [25,37,68].
Participates in PTGS or RNAi pathways	In all plants [12,69].	Limited participation in all plants [70,71].
sRNA-dependent activity	Uses microRNA precursors, 21–22nt, 24nt small RNAs [12].	sRNA-independent [72].
*de novo* DNA methylation	Yes, using 21–22nt siRNAs [12].	Not involved in *de novo* DNA methylation [12].
Maintenance DNA methylation	Yes, in all sequence contexts [12].	Yes, in all sequence contexts [22].
Silenced TEs	Short TEs, the edges of long TEs, euchromatic TEs and TE relics [11].	The bodies of long TEs, heterochromatic TEs, protein-coding TEs and mobile TEs [11].
Dominance	Euchromatin [11,73].	Heterochromatin [11,22].
Gene body silencing	Common [12].	Rare [22].
Pol IV activity	Yes [12].	No [12].
Argonaute and Dicer activity	Yes, AGO1, AGO4, AGO6 and AGO9. Complete or partial Dicer activity with DCL1, DCL2, DCL3 and DCL4 depending on the small RNA type, RdDM component involved and target loci [12,74,75].	No [12].
Nucleosome displacement	No, but involved in DNA methylation [12].	Yes, involved in DNA methylation, histone methylation, nucleosome positioning and other chromatin modifications [11,22,76].

This table summarises the features distinguishing the epigenetic modifications directly controlled by the DDM1 and RdDM pathways.

## Data Availability

Not applicable.
